# Mangrove Conservation in Macao SAR, China: The Role of Environmental Education among School Students

**DOI:** 10.3390/ijerph19063147

**Published:** 2022-03-08

**Authors:** Karen Araño Tagulao, Allan B. I. Bernardo, Loi Hoi Kei, Cristina Sousa Coutinho Calheiros

**Affiliations:** 1 Institute of Science and Environment, University of St. Joseph, Rua de Londres 106, Macao SAR 999078, China; 201800417@usj.edu.mo (L.H.K.); cristina@calheiros.org (C.S.C.C.); 2 Department of Psychology, De La Salle University, 2401 Taft Avenue, Manila 1004, Philippines; allan.bernardo@dlsu.edu.ph; 3 Interdisciplinary Centre of Marine and Environmental Research (CIIMAR/CIMAR), University of Porto, 4450–208 Matosinhos, Portugal

**Keywords:** conservation, urban mangroves, environmental orientation, environmental protection, NEP-New Ecological Paradigm

## Abstract

Mangrove forests are one of the most ecologically valuable ecosystems in the world and provide a wide variety of ecosystem services to coastal communities, including cities. Macao, a highly urbanized coastal city located on the southern coast of China west of the Pearl River, is home to several species of mangroves with many associated flora and fauna. Mangrove forests in Macao are vulnerable to threats due to pressure from rapid and massive urban developments in the area, which led to mangrove loss in the past decades. To address this issue, the local authorities established special Ecological Zones for the management of the local mangroves. To reinforce local conservation efforts, educating the local population about the value of mangroves, especially school students, is of utmost importance. To evaluate the impact of environmental education activities on the environmental orientation, knowledge, and values of students toward mangrove conservation in Macao, a quasi-experimental study was undertaken. The effectiveness of a mangroves exhibition and field visit were evaluated using the New Environmental Paradigm (NEP) Scale—Macao version in a group of local school students who participated in the activities. Overall, the results provided consistently positive evaluations of the impact of the environmental education program. The strongest improvements were found in the students’ pro-environmental orientations, knowledge about mangroves, and value for environmental protection.

## 1. Introduction

Mangrove forests are one of the most ecologically valuable and biologically diverse coastal ecosystems in the world, providing a wide range of ecosystem services on a local, regional, and global scale [1,2]. Recently, the role of mangroves in carbon sequestration has been strongly promoted, and mangroves are now on the international climate mitigation and adaptation agenda [3]. Studies related to storm protection, erosion control, and water pollution treatment have increased the estimated values of these systems to an average of 21,100 USD/ha/year [1,4]. Moreover, mangroves provide intangible but equally important cultural ecosystem services, such as recreation and tourism [5] and spiritual [6] and scientific value [7].

While most studies on mangroves have been in areas with highly dependent coastal rural communities, urban mangroves (mangroves in and around cities) are attracting more attention as possible solutions to environmental issues that go hand in hand with rapid urbanization [3,8]. They are important in biodiversity preservation and maintenance [9]; coastline stabilization and the protection of coastal cities from storm damage—especially important due to more frequent extreme weather events [5,10]; carbon capture and storage [11]; and the provision of highly valued cultural services in cities, such as recreation and education [5,12].

Despite their value, mangroves are often marginalized and degraded across much of the tropics [13]. The main drivers of recent loss are anthropogenic threats such as pollution, overextraction, and the conversion of mangrove forests to aquaculture [3,14]. Urban development in coastal zones is also causing mangrove degradation in specific places, such as China and Vietnam [3,14,15,16,17]. Large areas of mangrove forests and associated mudflats have been converted into infrastructure and urban developments in this part of the world. Urban expansion (increase in the area of built-up land) was negatively correlated with the change in mangrove forest area in Guangdong Province between 2005 and 2015 [18]. Similar scenarios were reported in other Southeast Asian countries such as the Gulf of Thailand and southern Malaysia and Singapore, wherein urbanization-driven deforestation is aggravated by the proximity of growing coastal populations to the mangrove forests [19].

Though deforestation rates declined in the past decade, the future of mangroves remains uncertain [14], especially in coastal urban areas. Information failure further aggravates mangrove loss due to the local people’s lack of awareness about the conservation value of mangrove ecosystems [20,21]. This reflects a lack of understanding of the direct relationship between mangrove ecosystems and the benefits they provide to humans [20]. This may be more prevalent in coastal urban areas, where the human population is growing. Despite their proximity to a mangrove forest, people may not realize the benefits of this important ecosystem because reliance on the forest in terms of direct goods and services is not obvious compared with living in coastal rural areas.

Macao, a highly urbanized coastal city situated on the west shore of the Pearl River Delta in southern China, is home to several species of mangroves and many associated flora and fauna. The area consisting of mangroves in the territory was estimated to be approximately 60 ha in the 1970s [22]. In 2003, the Macao SAR government established Ecological Zones (managed zones), in which 40 ha are mainly mangroves. Land reclamation and massive construction projects have reduced the mangroves’ area to approximately 19 ha [23]. The establishment of Ecological Zones and other efforts of local authorities and the community may have led to a slight increase in the forest cover in the past few years. However, more strategies are needed to promote the conservation of the local mangroves. One possible solution to this issue is to positively change our attitude and behavior toward the environment, i.e., the mangroves, through environmental education.

Environmental education impacts the behavior of students in several aspects, such as enhancing their literacy about ecosystems and influencing their values, attitudes, and willingness to be active in the conservation of nature [24]. Students who are likely to participate in ‘light green’ activities (recycling, saving water and electricity, using public transportation, etc.) require minor changes to their habits. They are more likely to engage in typical activities that require little effort and inconvenience, such as switching off unused electrical appliances and recycling [25]. Environmental education has been increasingly incorporated into the curriculum of Macao, but no specific activities are being implemented related to mangrove conservation. While some higher education institutions and local industries are incorporating the UN Sustainable Development Goals (SDGs) in their agenda, there is no clear program related to this in schools. The Institute of Science and Environment of the University of St. Joseph in Macao recognizes the need to take action to achieve the SDGs, especially SDG 14, Life below water, by offering environmental education activities specific to mangroves. This study aimed to evaluate the impact of environmental education activities (exhibition visit and field trips to mangrove sites) on the environmental orientation, knowledge, and values of students toward mangrove conservation in Macao.

## 2. Materials and Methods

### 2.1. Environmental Education Activity

**Figure 1:**
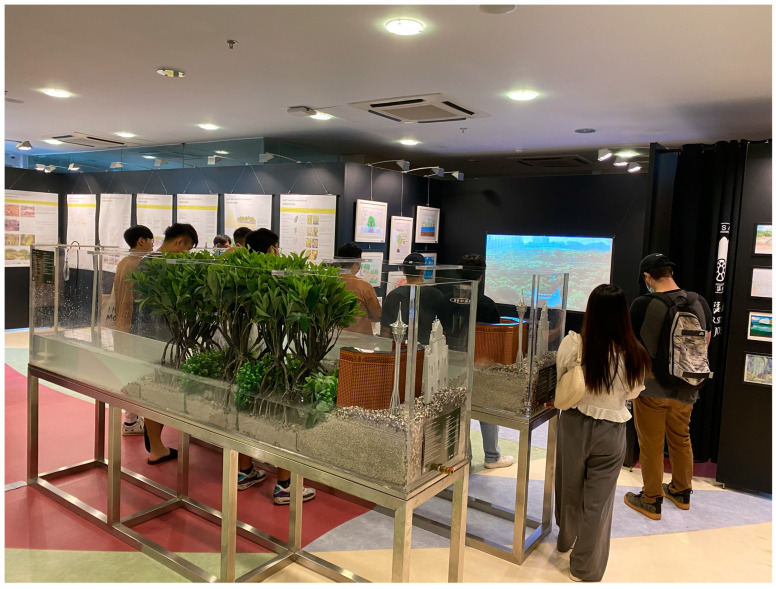
Students viewing the mangroves-related materials at the exhibition gallery.

**Figure 2:**
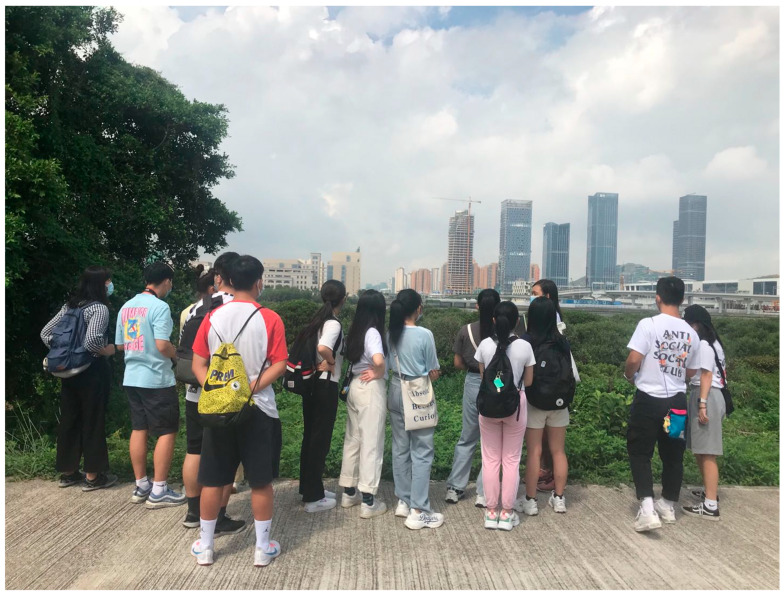
Guided visit to the local mangrove site.

A quasi-experimental study was conducted within the framework of environmental education activities implemented by the University of St. Joseph’s (Macao SAR) outreach programs. One of these activities was the mangroves exhibition (http://ise.usj.edu.mo/research/projects/macaos-mangroves-a-coastal-treasure-exhibition, accessed on 21 February 2022) held during 1–30 September 2020 at the Kent Wong Exhibition Gallery, University of Saint Joseph. The exhibition showcased the local mangrove ecosystem that makes up an essential part of Macao’s natural environment through scientific display boards, photographs, films, and interactive tank models (Figure 1). The scientific display boards provided essential information about mangroves such as: (1) What are mangroves? (2) Why are mangroves unique? (3) Where can we find mangroves? (4) Why are mangroves important? (5) What threats do mangrove face? (6) What can we do for the mangroves? High quality photographs showcasing not only the uniqueness and beauty of the mangrove flora but also the various fauna—fishes, crabs, and birds—were also displayed. The interactive tank demonstrated the ability of mangroves to attenuate waves. A 10 min video (https://youtu.be/-hHcwtXcazA, accessed on 21 February 2022) about the local mangroves was also shown to the students in the exhibition gallery. The exhibition was complemented by guided field visits to the Ecological Zone (22°08′29.0″ N 113°33′07.2″ E, https://www.dspa.gov.mo/place3.aspx, accessed on 21 February 2022) in Macao, an important local mangrove site (on the same day as the exhibition visit) with the aim to raise community awareness and curiosity and encourage people to cherish this valuable habitat. This visit comprised a guided walk through the forest, highlighting its uniqueness, important ecosystem services, and the threats mangroves face (Figure 2).

The exhibition tour and mangrove site visits for this school were organized in groups of 30–35 students between 14 to 24 September 2020. The students were given 45 min to explore the exhibition, followed by 1 h of field visit, with explanations given by mangrove experts from the university.

### 2.2. Participants

To evaluate the effectiveness of the aforementioned activities, participants were requested to do a pre- and post-survey questionnaire the day before and the day after the activity, respectively. The participants considered for this study were students from Grades 7 to 12 (ages 12 to 19 years) who were all from the same local school. This school is among the ones that accepted the invitation to implement the mangroves environmental education program and has students who were willing to participate in the survey. The students did not have prior knowledge provided by their teachers concerning the exhibition or the field trips. The students who participated in the survey were those who also attended both the exhibition visit and the field trips. A total of 83 participants, of whom 38 were female and 45 were male, completed the survey. They visited the exhibition and joined the field trips within the time schedule of their classes and were accompanied by 2 teachers.

### 2.3. Measures

To assess the effectiveness of the environmental education program, several measures were used to assess changes in the participants’ environmental orientations, knowledge, and values.

*New Environmental Paradigm (NEP) Scale—Macao version.* The scale developed by Dunlap et al. [26] was adapted to the Macao context. The items in the original scale were statements expressing how a specific relationship exists between humans and the environment. In the adapted scale, some of the items were revised to specifically refer to mangroves, an important environmental feature of the Macao ecology (see Table 1 for the adapted items). Students were asked to indicate their degree of agreement for each of the items using a Likert scale survey (Appendix A) from 1 (strongly disagree) to 5 (strongly agree). The items identified by Dunlap et al. [26] as reverse scored were reversed before the average score was computed. A higher mean indicated stronger pro-environmental orientation.

The NEP scale has been used by social–psychological models to address people’s environmental concerns, attitudes, and behaviors, and is a well-established measure of pro-environmental orientation [26,27]. However, the assessment of the internal consistency of the NEP-Macao version was not adequate (Cronbach’s α = 0.50, which was below the criterion of 0.70). Therefore, we examined the item–total correlations and removed four items (i.e., items numbered 4, 6, 8, and 10 in Table 1) that had low and non-significant correlations with the total; the reduction of items in the original NEP has been done in similar studies that aimed to improve the internal consistency of the scale [28,29]. The 11-item version of the NEP-Macao had good internal consistency; for the pre-test, Cronbach’s α = 0.72, and for the post-test, Cronbach’s α = 0.75.

*Knowledge and attitudes questionnaire.* Five additional items were constructed to inquire into the students’ other knowledge and attitudes separate from the scope of the NEP-Macao version. These items inquired into the students’ current knowledge, exhibition knowledge, environmental connection, environmental value, and environmental protection (see Table 2 for the items). Students were also asked to indicate their agreement using the same scale as the NEP-Macao version.

### 2.4. Statistical Analysis

Descriptive statistics, repeated measures analysis of covariance (ANCOVA), and multivariate analysis of covariance (MANCOVA) were conducted to analyze the NEP-Macao scores and the answers to the knowledge and attitudes questionnaire before and after the environmental education activity.

The ANCOVA is a commonly used statistical method for comparing at least two sets of scores; the repeated measures ANCOVA is used to compare at least two sets of related scores from the same sample while also assessing the effects of covariates. In this study, the pre- and post-test scores of the participants were repeated measures of the same variable; the ANCOVA evaluated whether there were statistically significant changes between the pre- and post-test scores, while statistically controlling two covariates: age and gender of the participants.

The MANCOVA is a variation on the ANCOVA that is used when several scores that are assumed to be correlated with each other are compared simultaneously. MANCOVA was used to evaluate whether there were changes in the five knowledge and attitudes items from pre-test to post-test, while statistically controlling two covariates: age and gender of the participants.

Prior to conducting the analyses, we tested some assumptions regarding the normality of the NEP pre- and post-test data. Normality refers to whether the data for each sample are distributed normally, and the Shapiro–Wilk test for normality indicated that the data were significantly different from a normal distribution (W = 0.931, *p* < 0.001 for the pre-test NEP scores; W = 0.826, *p* < 0.001 for the post-test NEP scores). The normal Q–Q plots for the two sets of scores indicated 5 outliers. Removing these 5 outliers indicated that the normality assumptions were met (new Shapiro–Wilk: W = 0.975, *p* = 0.128 for the pre-test NEP scores; W = 0.976, *p* = 0.156 for the post-test NEP scores). This result indicated that it was safe to do the ANCOVA on the NEP scores without the outliers. All analyses reported were conducted using SPSS software (IBM SPSS Statistics for Apple MacOS, Version 24.0, IBM Corp, Armonk, NY, USA).

## 3. Results

In Table 3, the sociodemographic characteristics of respondents who were included in this study are presented.

We first looked at the NEP-Macao scores, and the basic statistics are summarized in Table 4. An ocular inspection of the means in the pre-test and post-test suggested that there were increases in most measures, and these increases were verified in the ANCOVA for repeated measures.

The ANCOVA provided useful results. First, the main effect of the environmental education activity was statistically significant; there was a significant increase from pre- to post-test (*F*(1, 75) = 6.029, *p* = 0.016, partial η^2^ = 0.074), although the effect size was small. We inferred that participation in the activity resulted in improved environmental orientation among the students using a well-established measure adapted for the Macao context. The ANCOVA also provided results related to the covariates (age and gender) and how these interacted with the environmental education activity. Gender had no effect on the NEP-Macao scores (*F*(1, 75) = 0.773, *p* = 0.382, partial η^2^ = 0.010), and gender did not interact with the treatment effects either (*F*(1, 75) = 0.477, *p* = 0.492, partial η^2^ = 0.006). Age was associated with the NEP-Macao scores (*F*(1, 75) = 6.657, *p* = 0.016 partial η^2^ = 0.082); older students had higher environmental orientations compared with younger students. However, the effect of the environmental education activity was the same across all ages (*F*(1, 75) = 3.209, *p* = 0.077, partial η^2^ = 0.041). In summary, there was a small but statistically significant improvement in the students’ environmental orientation after engaging in the environmental exhibit, and this improvement was not affected by the students’ age or gender.

As regards the students’ knowledge about mangroves, the descriptive statistics are summarized in Table 5. The pre-test scores indicated that participants did not report high environmental knowledge before the activity. The MANCOVA provided several useful tests, the first of which was the main effect of the environmental education activity; the results indicated a significant increase after the activity, *F*(1, 75) = 4.084, *p* = 0.047, partial η^2^ = 0.052; the effect size was small but statistically significant.

The MANCOVA provided results related to differences in the type of environmental knowledge or belief and their interactions with the environmental education activity, but there was no statistically significant interaction between the effect of the treatment and the type of environmental knowledge or belief (*F*(4, 75) = 1.219, *p* = 0.303, partial η^2^ = 0.016). As shown in Table 5, the students’ knowledge and beliefs related to mangroves improved after the environmental education activity, and the improvement was consistent across the types of knowledge or beliefs. Finally, the MANCOVA provided results related to the covariates (age and gender) and their interactions with the environmental education activity, but the results indicated that neither age nor gender interacted with the effects of the environmental education intervention on any of the types of environmental knowledge or beliefs (i.e., for all interaction effects, *F*-scores were non-significant). This meant that the effect of the environmental education activity was similar regardless of age and gender.

Overall, the results provided consistently positive evaluations of the impact of the environmental education activity. Although the statistical effect sizes were small, there was a consistent statistically significant improvement in the students’ pro-environmental orientations, knowledge about mangroves, and value for environmental protection.

## 4. Discussion

This study revealed positive outcomes of environmental education interventions as reflected in the improvement in the general environmental orientation among students who participated in the activities. Environmental education has been increasingly incorporated in the educational curriculum of Macao, and schools are required to carry out a curriculum reform based on a prescribed framework with their own characteristics and education. However, there is no standard assessment of the students’ understanding toward environment-related topics. The improvement in environmental orientation shown in our study suggests the effectiveness of non-formal environmental education activities such as exhibitions and field trips to improve environmental orientation. Environmental education was shown to impact the behavior of students in several aspects, such as enhancing their literacy about ecosystems and influencing their values, attitudes, and willingness to be active in the conservation of nature [24]. We aimed to enhance students’ environmental knowledge and environmental attitudes. Environmental knowledge implies a person’s knowledge and awareness about environmental problems and their possible solutions and increasing their concern and awareness, but it does not necessarily result in behavioral change [30]. Environmental attitudes assess the environmentally aware lifestyle, consumption habits, and solutions to environmental problems of an individual. According to Bamberg et al. [30], environmental knowledge and attitudes are interconnected. They strengthen each other, especially in information seeking about environmental issues.

Teaching via school curricula is one of the traditional ways of promoting pro-environmental behavior and knowledge. Learning through participation (hands-on experiences) or learning through ‘knowing eye’ (visual literacy)—similar to the exhibition visit and field trips conducted in our study—are less directly observable and more implicit methods. Taylor and Enggass [31] suggested that once we read an environment, we cultivate a knowing eye. Individuals may gain visual literacy through reading, seeing, deeply perceiving, and critically analyzing the physical environment. Thus, the physical environment is described as the ‘three-dimensional textbook’ or ‘silent curriculum’ [31]. The natural environment acts as an effective learning arena for children [32]. Moreover, educational field trips show a positive cognitive and affective effect [33]. Participation in nature-based learning is more related to environmental behavior than environmental knowledge [34]. A well-planned field trip with a follow-up allows students to develop their knowledge and skills that add value to their everyday classroom experiences [35]. Participation in field trips for grade school students shows a substantial increase in ecosystem knowledge [36].

With regards to the students’ knowledge about mangroves, the pre-test scores indicated that participants did not have high knowledge about mangroves before the activity. This is an indication of a lack of integration in the local curriculum of concepts related to the local ecosystems, i.e., the mangroves, and a lack of positive reinforcement outside of schools for mangroves awareness. We also observed this in the interactions between the mangroves project team and the general community during the community outreach activities (field trips, talks, and exhibition). Information failure aggravates mangrove loss as a consequence of the local people’s lack of awareness about the conservation value of mangrove ecosystems [20,21]. This reflects a lack of awareness and understanding of the direct relationship between mangrove ecosystems and the benefits they provide to humans as some benefits are off-site and not acknowledged as being related to mangroves [20]. Although the benefits are not obvious to the local population as they are not reliant on fisheries or harvests directly taken from the mangroves, there are many ecosystem services that mangroves provide to the city and its people. The forest cover is small (approximately 19 ha in 2020), but mangroves in Macao significantly contribute to local biodiversity and provide other important ecosystem services as they serve as coastal nursery habitats for fishes and other marine life, contribute to carbon sequestration and storage, filter and improve water quality, act as a buffer zone between land and sea, and provide sites for ecotourism and education [37,38]. However, the challenge is how to promote this idea to the local community to create an impact and foster more conservation efforts. The results from our study indicate that environmental education activities may be a good solution to the lack of awareness about mangroves in the city. The students strongly agreed with the proposition that exhibitions can help them know more about mangroves. Activities such as these should be implemented more often for students and the public.

A similar study in Malaysia reported that an environmental education program, focused on ecosystem services, did not have an effect on students’ awareness and knowledge about mangroves [39]. Moreover, while student respondents were not familiar with the cultural services provided by mangroves in Setiu Wetlands, they had existing knowledge about mangroves as some lived in close proximity to the ecosystem. Their findings suggested that students do not see a clear link between their lives and mangroves. This is an important point to consider in the Macao scenario. It is essential to account for the value of intangible ecosystem (cultural) services in future education programs.

The perception of mangroves by the public is essential for conservation [40], and it is important to showcase them to the local community through various platforms. The use of high-quality, charismatic images of flagship species [41] on social media is a good strategy to promote the special features and conservation importance of such species and raise awareness [42]. As this study already has promotional materials, the selection of a possible flagship species should be conducted for use in further promoting the mangroves through various social media platforms. This may further reinforce our environmental education program.

Increased awareness about the ecosystem services provided by mangroves in Guangdong in southern China led to the establishment of nature reserves and the gradual expansion of the mangrove forest in recent years [17]. In Singapore, taking into account space limitation, the following opportunities were identified for the rehabilitation of urban mangroves: (1) identifying new areas of rehabilitation, (2) ecological engineering of new habitats, and (3) setting legal standards for habitat replacement corporate social responsibility to fund future rehabilitation [3]. A study in China proposed the following strategies to protect and manage the mangroves: publicity and education, resource restoration and protection technology, and legal system establishment and comprehensive protection and management [43].

The positive outcomes from this study may support decision makers at the level of governance to integrate similar activities for ecosystem conservation and habitat protection. Future research should explore other types of activities (mangrove planting, talks) with a broader audience (children to adults). A longer-term study, to evaluate the long-term impacts of such activities, should be conducted in parallel with monitoring the state of the mangrove forests in Macao. Environmental education combined with other mangrove conservation strategies similar to those practiced in other countries may also be adapted in Macao.

Furthermore, this study showed that the NEP scale is a useful research instrument to evaluate the effectiveness of environmental education activities on the local students’ environmental orientations and attitudes. Many other studies, involving primary school [44,45] to university students [46,47], determined impacts of interventions on ecological and environmental views of participants using the NEP. The outcomes from the present study indicate that this instrument can be used for similar studies in the future, although adjustments may be necessary depending on the variables involved.

## 5. Conclusions

Due to the benefits that mangroves provide to Macao, it is important to identify specific strategies that effectively promote awareness and conservation of the local mangroves and the general environment. This study provided insights on methodologies that can be applied to support the role of environmental education among school students related to mangrove conservation. The results of this study provided consistently positive evaluations of the impact of a mangroves exhibition and field trips to the forest. The strongest improvements were found in the students’ pro-environmental orientations, knowledge about mangroves, and value for environmental protection. Although the limitation of this study was the sample size, it suggests that it is possible to develop activities to reinforce local support for the sustainable management and conservation of mangroves. Findings from this study, although not limited to the following SDGs, are especially related to SDG 11, Sustainable cities and communities; SDG 12, Quality education; SDG 13, Climate action; and SDG 14, Life below water by offering high-quality environmental educational activities and educational resources specific to mangroves’ roles and service provision.

Future work should comprise a long-term study with a broader target population, including students from different school levels (primary, college and university) and adults that represent various sectors of society. Specific ecosystem services should be considered in future studies, especially the more intangible cultural services. The development of activities in classrooms aligned with outdoor field trips and associated methodologies combined with mangrove conservation strategies would broaden the approach toward improving environmental education on this subject. Integration of such activities into the formal education curricula in schools is recommended for policy makers to consider in the local education sector.

## Figures and Tables

**Table 1:** Revised *New Ecological Paradigm Scale—Macao Version* (adapted from [26]).

1. We are approaching the limit of the number of people the earth can support.
2. Humans have the right to modify the mangrove environment to suit their needs.
3. When humans interfere with mangroves, it often produces disastrous consequences.
4. Human ingenuity will ensure that we do not make the earth unlivable.
5. Humans are severely abusing the mangroves.
6. The mangrove ecosystem has plenty of natural resources if we just learn how to develop them.
7. Plants and animals in mangroves have as much right as humans to exist.
8. The balance of nature in the mangrove ecosystem is strong enough to cope with the impacts of modern industrial nations.
9. Despite our special abilities, humans are still subject to the laws of nature.
10. The mangrove crisis has been greatly exaggerated.
11. The earth is like a spaceship with very limited room and resources.
12. Humans were meant to rule over the rest of nature.
13. The balance of nature in mangroves is very delicate and easily upset.
14. Humans will eventually learn enough about how nature works to be able to control it.
15. If things continue on their present course, we will soon experience a major environmental catastrophe.

**Table 2:** Items on knowledge and attitudes.

Knowledge and Attitudes Dimension	Items
Environmental knowledge	I already know about mangroves.
Exhibition knowledge	Exhibition visits help me understand more about mangroves.
Environmental connection	I feel more connected with nature when classes are held in outdoor spaces.
Environmental value	Wetlands, like mangroves, are important for coastal cities like Macao.
Environmental protection	Mangroves should be protected and conserved.

**Table 3:** Sociodemographic characteristics of respondents.

Description	Categories	Frequency
Gender	Male	45
	Female	38
Age (years)	12	18
	13	22
	14	5
	15	10
	16	9
	17	15
	18	3
	19	1
Grade Level	7	18
	8	31
	9	1
	10	6
	11	18
	12	9

**Table 4:** Summary of descriptive statistics for NEP-Macao items (*n* = 78).

	Pre-Test				Post-Test				Change
Measure	*M*	*SD*	Skewness	Kurtosis	*M*	*SD*	Skewness	Kurtosis	
NEP-Macao (complete scale)	3.589	0.365	0.225	−0.380	3.91	0.29	0.067	−0.161	0.321
NEP Item 1	3.744	0.797	−0.292	−0.228	4.154	0.722	−1.728	5.878	0.041
NEP Item 2	2.923	1.003	0.316	−0.648	3.295	1.186	−0.646	−0.923	0.372
NEP Item 3	3.769	0.719	0.378	−0.982	4.321	0.497	0.454	−1.014	0.552
NEP Item 5	3.641	0.805	−0.321	0.549	4.026	0.789	−1.022	1.357	0.385
NEP Item 7	4.077	0.864	−0.894	1.035	4.577	0.57	−1.388	3.376	0.5
NEP Item 9	3.936	0.671	−0.190	−0.003	4.167	0.59	−0.439	1.618	0.231
NEP Item 11	3.859	0.936	−0.589	−0.404	4.154	0.927	−1.319	1.655	0.259
NEP Item 12	3.564	1.135	−0.327	−0.924	3.769	1.127	−0.868	−0.126	0.205
NEP Item 13	3.449	0.696	0.069	−0.149	3.782	0.595	0.104	−0.385	0.333
NEP Item 14	2.59	0.904	0.7	−0.048	2.551	0.935	0.63	−0.108	−0.039
NEP Item 15	3.923	0.923	−0.659	0.207	4.256	0.612	−0.553	1.277	0.333

**Table 5:** Summary of descriptive statistics for environmental knowledge and beliefs (*n* = 78).

	Pre-Test				Post-Test				Change
Measure	*M*	*SD*	Skewness	Kurtosis	*M*	*SD*	Skewness	Kurtosis	
Environmental knowledge	2.885	1.006	−0.234	−0.594	3.897	0.847	−2.174	5.735	1.012
Exhibition knowledge	3.949	0.754	−0.101	−0.749	4.513	0.528	−0.323	−1.311	0.564
Environmental connection	3.769	0.896	−0.856	1.131	4.154	0.994	−1.781	3.482	0.385
Environmental value	3.692	0.708	0.072	−0.345	4.449	0.501	0.21	−2.008	0.757
Environmental protection	4.308	0.726	−0.757	0.059	4.654	0.505	−0.967	−0.363	0.346

## Data Availability

The data presented in this study are available on request from the corresponding author.

## Appendix A. Survey Questionnaire

Kindly help us answer the following questions. Your identity will be anonymous. The results of this survey will be used for education/scientific research only. Thank you!

**Table A1:** Survey Questionnaire.

Age:				
Gender:				
Grade Level:				
1. We are approaching the limit of the number of people the earth can support.				
Strongly Agree	Agree	Not Sure	Disagree	Strongly Disagree
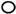
2. Humans have the right to modify the mangrove environment to suit their needs.				
Strongly Agree	Agree	Not Sure	Disagree	Strongly Disagree
3. When humans interfere with mangroves, it often produces disastrous consequences.				
Strongly Agree	Agree	Not Sure	Disagree	Strongly Disagree
4. Human ingenuity will ensure that we do not make the earth unlivable.				
Strongly Agree	Agree	Not Sure	Disagree	Strongly Disagree
5. Humans are severely abusing the mangroves.				
Strongly Agree	Agree	Not Sure	Disagree	Strongly Disagree
6. The mangrove ecosystem has plenty of natural resources if we just learn how to develop them.				
Strongly Agree	Agree	Not Sure	Disagree	Strongly Disagree
7. Plants and animals in mangroves have as much right as humans to exist.				
Strongly Agree	Agree	Not Sure	Disagree	Strongly Disagree
8. The balance of nature in the mangrove ecosystem is strong enough to cope with the impacts of modern industrial nations.				
Strongly Agree	Agree	Not Sure	Disagree	Strongly Disagree
9. Despite our special abilities, humans are still subject to the laws of nature.				
Strongly Agree	Agree	Not Sure	Disagree	Strongly Disagree
10. The mangrove crisis has been greatly exaggerated.				
Strongly Agree	Agree	Not Sure	Disagree	Strongly Disagree
11. The earth is like a spaceship with very limited room and resources.				
Strongly Agree	Agree	Not Sure	Disagree	Strongly Disagree
12. Humans were meant to rule over the rest of nature.				
Strongly Agree	Agree	Not Sure	Disagree	Strongly Disagree
13. The balance of nature in mangroves is very delicate and easily upset.				
Strongly Agree	Agree	Not Sure	Disagree	Strongly Disagree
14. Humans will eventually learn enough about how nature works to be able to control it.				
Strongly Agree	Agree	Not Sure	Disagree	Strongly Disagree
15. If things continue on their present course, we will soon experience a major environmental catastrophe.				
Strongly Agree	Agree	Not Sure	Disagree	Strongly Disagree
16. I already know about mangroves.				
Strongly Agree	Agree	Not Sure	Disagree	Strongly Disagree
17. Exhibition visits help me understand more about mangroves.				
Strongly Agree	Agree	Not Sure	Disagree	Strongly Disagree
18. I feel more connected with nature when classes are held in outdoor spaces.				
Strongly Agree	Agree	Not Sure	Disagree	Strongly Disagree
19. Wetlands, like mangroves, are important for coastal cities like Macao.				
Strongly Agree	Agree	Not Sure	Disagree	Strongly Disagree
20. Mangroves should be protected and conserved.				
Strongly Agree	Agree	Not Sure	Disagree	Strongly Disagree
